# Anatomical Location Is Associated with Clinicopathological Features and Long-Term Oncological Outcomes in Mucinous Colorectal Adenocarcinoma: A Population-Based Analysis of 40,698 Patients from the SEER Database

**DOI:** 10.3390/jcm15145584

**Published:** 2026-07-16

**Authors:** Burak Kutlu, Çiğdem Benlice

**Affiliations:** 1 General Surgery, Acıbadem Ankara Hospital, 06450 Ankara, Turkey; 2 General Surgery, Istanbul Health and Technology University, 34275 Istanbul, Turkey; cigdem.benlice@acibadem.com

**Keywords:** mucinous adenocarcinoma, colorectal cancer, tumor location, cancer-specific survival

## Abstract

**Background**: Mucinous adenocarcinoma (MAC) of the colorectum is a biologically distinct histological subtype whose prognostic significance may vary substantially according to primary tumor location. The impact of anatomical site on clinicopathological characteristics and long-term survival in MAC remains incompletely characterized. This study aimed to evaluate the influence of tumor location on oncological outcomes in patients with mucinous colorectal cancer using a large population-based dataset. **Methods**: Patients diagnosed with mucinous colorectal adenocarcinoma between 2000 and 2023 were identified from the Surveillance, Epidemiology, and End Results (SEER) database. Operated patients were stratified by anatomical location into three groups: right colon (cecum, ascending colon, hepatic flexure, transverse colon), left colon (splenic flexure, descending colon, sigmoid colon, rectosigmoid junction), and rectum. The primary endpoints were overall survival (OS) and cancer-specific survival (CSS). Survival analyses were performed using the Kaplan–Meier method with log-rank testing. Multivariable Cox proportional hazards regression was used to assess the independent association of tumor location with survival outcomes, adjusting for age at diagnosis, year of diagnosis, sex, AJCC (American Joint Committee on Cancer) stage, tumor grade, receipt of radiotherapy and chemotherapy. Temporal trends in survival were evaluated across four consecutive diagnostic periods: 2000–2005, 2006–2011, 2012–2017, and 2018–2023. **Results**: A total of 40,698 patients with mucinous colorectal cancer were identified, of whom 40,174 underwent cancer-directed surgery (right colon *n* = 25,317; left colon *n* = 10,408; rectum *n* = 4449). The right colon was the predominant site of disease (62.9%). Median age was highest in the right colon group (74.0 years) and lowest in the rectum (65.0 years), while female sex predominated in right-sided tumors (55.7%) and male sex in rectal tumors (61.1%). Chemotherapy and radiotherapy utilization were markedly higher in the rectal group (68.6% and 64.5%, respectively) compared with the right colon (28.8% and 1.1%). Median OS was equivalent in the right and left colon groups (87.0 months each) but declined to 80.0 months in the rectal group. All pairwise CSS comparisons were statistically significant (all *p* < 0.001). On multivariable analysis, using the right colon as reference, the adjusted hazard ratios for CSS were 1.33 (95% CI: 1.28–1.39) for the left colon and 1.45 (95% CI: 1.34–1.57) for the rectum (both *p* < 0.001). Despite receiving the highest rates of multimodal therapy, rectal MAC demonstrated the worst long-term CSS across all anatomical groups. Temporal analyses revealed consistent CSS improvements in right-sided and left-sided MAC over the study period, whereas rectal MAC showed a non-linear trajectory with a plateau in the most recent diagnostic cohort (2018–2023). **Conclusions**: Mucinous colorectal adenocarcinoma demonstrates substantial biological and prognostic heterogeneity according to anatomical tumor location. Right-sided MAC was the most prevalent subtype and exhibited superior cancer-specific survival despite older patient age and lower treatment intensity. Rectal MAC demonstrated the worst long-term outcomes despite high utilization of multimodal neoadjuvant therapy, consistent with the established reduced responsiveness of mucinous tumors to conventional chemoradiotherapy. These findings underscore the necessity of incorporating anatomical location and tumor biology into individualized risk stratification and therapeutic planning for patients with mucinous colorectal cancer.

## 1. Introduction

Colorectal cancer (CRC) is the third most commonly diagnosed cancer worldwide, with its rising incidence largely attributed to lifestyle-related factors such as increased consumption of processed meats, reduced intake of fruits and vegetables, obesity, sedentary behavior, excessive alcohol use, and smoking [1]. Among the histological subtypes of CRC, mucinous adenocarcinoma (MAC) accounts for approximately 10–20% of all cases [2]. The World Health Organization defines MAC as a tumor in which more than 50% of the lesion is composed of pools of extracellular mucin containing malignant epithelium [3]. Compared to non-mucinous adenocarcinomas, MAC has been reported to predominantly affect younger patients, arise more frequently in the proximal colon, present at more advanced stages, and carry a poorer prognosis [4].

Despite the overall global rise in CRC incidence, the epidemiological trajectory of MAC has not been uniform across regions. In the United States, for instance, MAC incidence has declined substantially, from 4.5 per 100,000 individuals in 2000 to approximately 1.54 per 100,000 in 2018 [5]. This divergence underscores the importance of investigating MAC as a distinct entity with its own epidemiological, clinical, and biological characteristics.

MAC has attracted considerable research interest due to its distinctive clinicopathological and epidemiological profile, including unique demographic patterns, tumor behavior, and prognostic outcomes that differ from those of non-mucinous CRC [6]. Recent molecular studies have further demonstrated that MACs harbor distinct genetic backgrounds and clinicopathological features, including a higher frequency of microsatellite instability, when compared to non-mucinous CRCs [7].

The prognostic significance of primary tumor location in CRC has garnered considerable attention in recent years. Left-sided and right-sided tumors are recognized to differ substantially in terms of embryological origin, genetic carcinogenic pathways, and clinical outcomes. The right-sided colon derives embryologically from the midgut, whereas the left-sided colon and rectum originate from the hindgut. This embryological divergence is closely correlated with differences in molecular subtypes and carcinogenic mechanisms [8,9]. However, the specific impact of tumor location on the clinicopathological features and prognosis of MAC remains insufficiently characterized.

The aim of this study was to investigate the clinicopathological characteristics and long-term oncological outcomes of mucinous colorectal adenocarcinoma according to anatomical tumor location using the Surveillance, Epidemiology, and End Results (SEER) database, with particular emphasis on differences between right-sided colon, left-sided colon, and rectal tumors.

## 2. Methods

### 2.1. Data Source and Study Population

This study utilized data from the SEER program, a population-based cancer registry that captures demographic, clinicopathological, and survival data for cancer patients across geographically diverse regions of the United States (National Cancer Institute, Bethesda, MD, USA). The study population comprised patients diagnosed with mucinous colorectal adenocarcinoma between 2000 and 2023. Patients were initially stratified into two groups based on the recorded reason for cancer-directed surgery: those who underwent surgical resection (surgery performed) and those who did not. Among operated patients, cases were further classified by anatomical tumor location into three groups: right colon (cecum, ascending colon, hepatic flexure, and transverse colon), left colon (splenic flexure, descending colon, sigmoid colon, and rectosigmoid junction, and rectum. Anatomical sites were selected according to ICD-O-3 topography codes and categorized as right colon (C18.0, C18.2, C18.3, C18.4), left colon (C18.5, C18.6, C18.7, C19.9), and rectum (C20.9).

AJCC 6th edition stage grouping was used to maintain a consistent staging framework across the entire study period. Patients with AJCC (American Joint Committee on Cancer) stage IV disease were excluded from the primary analysis.

### 2.2. Outcome Definitions

Two survival endpoints were examined: overall survival (OS) and cancer-specific survival (CSS). OS was defined as the time from diagnosis to death from any cause, with living patients censored at the date of last contact. CSS was defined as the time from diagnosis to death attributable to CRC as recorded in the SEER cause-specific death classification, with deaths from other causes treated as censored observations.

### 2.3. Statistical Analysis

As this was a retrospective population-based registry study, no formal sample size calculation was performed. However, the large SEER cohort provided sufficient statistical power for the planned comparative and multivariable survival analyses.

Baseline demographic and clinicopathological characteristics were compared across groups using the chi-square test for categorical variables and the Kruskal–Wallis test for continuous variables. Categorical variables are reported as count and percentage within group; continuous variables are reported as median with interquartile range (IQR).

Survival analysis was performed using the Kaplan–Meier method, and survival curves were compared using the log-rank test. Survival estimates were reported at 12, 36, and 60 months. For the anatomical subgroup analysis, pairwise log-rank comparisons were conducted between right colon, left colon, and rectum groups.

Multivariable Cox proportional hazards regression was performed to assess the independent association of surgical status and tumor location with survival outcomes. Colon and rectal cancers were modeled separately. The following covariates were included in all models: age at diagnosis (per 10-year increment), year of diagnosis (per 5-year increment), sex, AJCC stage, tumor grade and receipt of radiotherapy and chemotherapy. Missing values in categorical covariates were retained as explicit categories to preserve sample size and avoid selection bias introduced by complete-case analysis. For the anatomical site comparison, the right colon was used as the reference category. Results are reported as adjusted hazard ratios (HR) with 95% confidence intervals (CI). As a sensitivity analysis for cancer-specific survival, Fine–Gray subdistribution hazard modelling was performed, treating colorectal cancer-specific death as the event of interest and death from other causes as a competing event.

To evaluate temporal trends in survival, operated patients were stratified into four consecutive diagnosis periods: 2000–2005, 2006–2011, 2012–2017, and 2018–2023. Kaplan–Meier survival estimates at 36 and 60 months were calculated for each period and plotted separately for right colon, left colon, and rectum. Median follow-up duration was calculated separately for each diagnostic period.

All statistical tests were two-sided, and a *p* value of less than 0.05 was considered statistically significant. Statistical analyses were performed using Python version 3.12.3 (Python Software Foundation, Beaverton, OR, USA) with the lifelines (version 0.30.0) and statsmodels (version 0.14.4) libraries. The proportional hazards assumption was assessed using Schoenfeld residuals. 

During the preparation of this manuscript, OpenAI ChatGPT (GPT-5.6 Thinking) and Anthropic Claude (Claude Sonnet 5) were used exclusively for language editing, grammar correction, manuscript structuring, improvement of readability, and assistance in drafting selected manuscript sections.

## 3. Results

**Figure 1:**
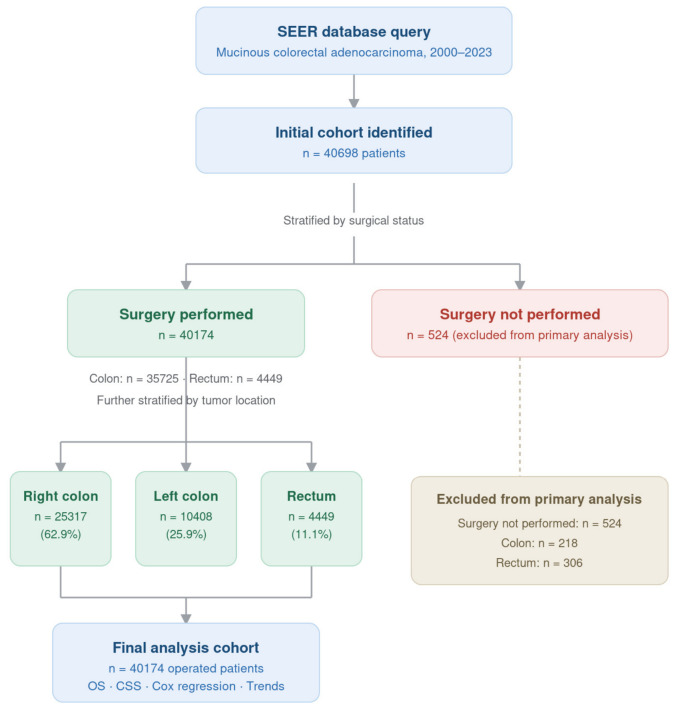
STROBE-compliant patient selection flowchart. **CSS**: cancer-specific survival; **OS**: overall survival.

A total of 40,698 patients with mucinous colorectal cancer were identified from the SEER database. The flowchart illustrates the stepwise identification and exclusion process applied to derive the final study cohort (Figure 1). In the colon cohort, 35,725 patients underwent cancer-directed surgery while 218 did not. In the rectal cohort, 4449 patients underwent surgery while 306 did not. Among operated colon patients, the median age was 72.0 years (IQR: 61.0–81.0), compared to 76.0 years (IQR: 64.0–85.0) in the non-operated group. In the rectal cohort, median ages were 65.0 years (IQR: 54.0–75.0) and 69.0 years (IQR: 55.2–80.0) for operated and non-operated patients, respectively.

Baseline demographic and clinicopathological characteristics of operated mucinous colorectal cancer patients according to tumor location are summarized in Table 1. Median age was highest in the right colon group at 74.0 years (IQR: 63.0–82.0), compared to 68.0 years (IQR: 57.0–78.0) in the left colon and 65.0 years (IQR: 54.0–75.0) in the rectum (*p* < 0.001). Regarding sex distribution, females predominated in the right colon group (55.7%), whereas males accounted for the majority of rectal cancer patients (61.1%). Treatment patterns also varied markedly by anatomical site. Chemotherapy utilization was highest in the rectal group (68.6%) and lowest in the right colon group (28.8%). Radiotherapy rates similarly followed an anatomical gradient, being substantially higher in the rectum (64.5%) compared to the left colon (7.9%) and right colon (1.1%) (*p* < 0.001).

**Figure 2:**
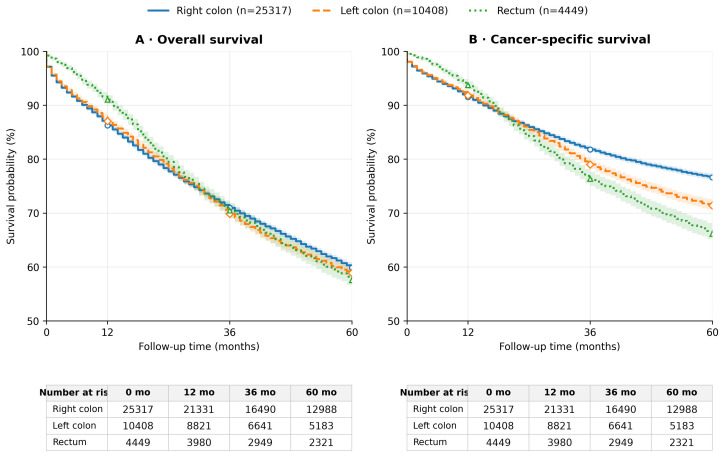
Kaplan–Meier survival estimates according to anatomical tumor location in operated patients with mucinous colorectal cancer. (**A**) Overall survival (OS) rates at 12, 36, and 60 months for right-sided colon, left-sided colon, and rectal cancer. (**B**) Cancer-specific survival (CSS) rates at 12, 36, and 60 months according to tumor location. Shaded areas indicate 95% confidence intervals. Numbers at risk shown below each panel.

Among operated patients, comparison of the right colon (*n* = 25,317), left colon (*n* = 10,408), and rectum (*n* = 4449) groups demonstrated a significant effect of anatomical site on survival outcomes (*p* < 0.001). Median OS was equivalent in the right and left colon groups at 87.0 months, but declined to 80.0 months in the rectal group. For OS, a significant difference was observed between the right and left colon groups (*p* < 0.001), and right colon and rectum groups (*p* = 0.004), but no significant difference was found between the left colon and rectum (*p* = 0.823). For CSS, all pairwise comparisons across the three anatomical groups reached statistical significance (all *p* < 0.001) (Figure 2).

Using the right colon as the reference, multivariable analysis demonstrated that both the left colon and rectum were associated with significantly higher hazard for OS and CSS. For OS, the adjusted HR was 1.14 (95% CI: 1.10–1.17) for the left colon and 1.19 (95% CI: 1.12–1.25) for the rectum. For CSS, the adjusted HR was 1.33 (95% CI: 1.28–1.39) for the left colon and 1.45 (95% CI: 1.34–1.57) for the rectum (Table 2).

Statistically significant deviations from proportionality were observed and sensitivity analyses were performed using Cox models stratified by AJCC stage, tumor grade, chemotherapy, and radiotherapy. According to sensitivity analyses, the adjusted hazard ratios for left-sided and rectal tumors were 1.13 and 1.18 for overall survival and 1.32 and 1.44 for cancer-specific survival, respectively, compared with right-sided tumors.

In the Fine–Gray competing-risk sensitivity analysis, left-sided tumors (adjusted sHR 1.29, 95% CI 1.24–1.35) and rectal tumors (adjusted sHR 1.43, 95% CI 1.32–1.55) remained associated with a higher subdistribution hazard of cancer-specific death compared with right-sided tumors.

Several covariates independently predicted survival across. Increasing age was consistently associated with worse OS and CSS in both anatomical sites; each additional decade of age conferred an HR of 1.66 (95% CI: 1.64–1.69) for colon OS and 1.43 (95% CI: 1.38–1.47) for rectal OS. Later diagnosis year was associated with modestly improved survival across all models (all *p* ≤ 0.001), reflecting temporal improvements in management. Male sex was independently associated with worse OS and CSS in the colon cohort (HR 1.31 and 1.21, respectively; both *p* < 0.001), whereas in the rectal cohort, female sex carried a small but significant survival advantage (HR 0.84 for OS and 0.87 for CSS; *p* < 0.001 and *p* = 0.005, respectively). AJCC stage was the strongest tumor-related prognostic factor; in the colon cohort, stage III disease conferred an HR of 1.56 for OS and 2.38 for CSS relative to stage II (both *p* < 0.001), while stage I was associated with significantly better outcomes (HR 0.83 for OS and 0.51 for CSS). In the rectal cohort, stage I retained the most favorable prognosis (HR 0.56 for OS and 0.42 for CSS vs. stage III; both *p* < 0.001) (Table 3).

### Temporal Trends in Survival Outcomes

The median follow-up durations for patients diagnosed during 2000–2005, 2006–2011, 2012–2017, and 2018–2023 were 79.0 months (IQR 26.0–190.0), 87.0 months (IQR 29.0–160.0), 76.0 months (IQR 29.0–103.0), and 26.0 months (IQR 11.0–46.0), respectively.

**Figure 3:**
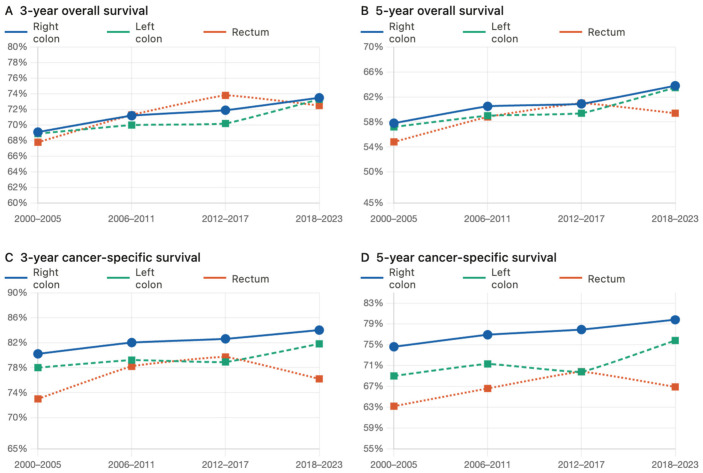
Temporal trends in survival outcomes by tumor location across four diagnosis periods (2000–2023). (**A**) 3-year overall survival; (**B**) 5-year overall survival; (**C**) 3-year cancer-specific survival; (**D**) 5-year cancer-specific survival. Survival estimates are presented for patients who underwent surgical resection, stratified by tumor location (right colon, left colon, and rectum) across four consecutive diagnosis periods: 2000–2005, 2006–2011, 2012–2017, and 2018–2023.

Three-year OS improved modestly across all three tumor locations over the study period. Right colon survival increased from 69.1% (2000–2005) to 73.5% (2018–2023), and left colon similarly rose from 68.5% to 73.3%. Rectal cancer demonstrated the most notable trajectory: after peaking at 73.8% in 2012–2017, it declined slightly to 72.5% in the most recent period, ultimately converging with colon cancer rates (Figure 3A). Five-year OS showed a comparable pattern. Right colon improved from 57.2% to 63.8%, and left colon from 57.2% to 63.5%. Rectal cancer, however, exhibited a distinctly different trend—rising from 54.8% to 61.0% between 2000 and 2017, but then declining to 59.1% in 2018–2023. Notably, in the earliest period rectal cancer had the lowest 5-year OS among the three groups, whereas by 2018–2023 it fell below both colon subgroups again after a transient improvement (Figure 3B).

Three-year CSS trends were more divergent by tumor location. Right colon demonstrated a consistent and steady improvement from 80.2% to 84.0%. Left colon remained relatively stable, ranging between 78.0% and 81.8%. In contrast, rectal cancer CSS rose substantially from 73.4% in 2000–2005 to 79.7% in 2012–2017, but declined to 76.9% in the most recent cohort (Figure 3C). Five-year CSS mirrored these trends. Right colon showed a consistent upward trajectory from 74.6% to 79.8%, and left colon improved from 69.0% to 75.8%. Rectal cancer again displayed a non-linear pattern, improving from 63.8% to 69.8% through 2012–2017 before plateauing at 66.9% in 2018–2023 (Figure 3D).

## 4. Discussion

In this large population-based analysis of patients with mucinous colorectal adenocarcinoma, we demonstrated that anatomical tumor location was strongly associated with distinct clinicopathological characteristics, treatment patterns, and long-term oncological outcomes. Several important findings emerged from the present study. MAC was predominantly localized in the right colon, supporting previous observations that mucinous tumors are more frequently associated with proximal colonic origin. Second, rectal MAC patients received substantially higher rates of chemotherapy and radiotherapy, yet still demonstrated inferior long-term survival outcomes compared with right-sided disease. Third, although rectal tumors tended to be diagnosed at relatively earlier T stages, their cancer-specific survival remained significantly worse. Finally, our findings support the concept that right- and left-sided mucinous colorectal cancers may represent biologically distinct disease entities, as suggested by previous molecular studies. These findings highlight the potential role of anatomical tumor location as an additional component of individualized risk assessment in mucinous colorectal adenocarcinoma.

Mucinous adenocarcinoma constitutes approximately 10–20% of colorectal cancers and is increasingly recognized as a distinct pathological and molecular subtype rather than merely a histological variant of conventional adenocarcinoma [10,11]. Previous studies have consistently shown that MAC demonstrates unique clinicopathological features, including younger age at presentation, advanced tumor stage, frequent peritoneal dissemination, and poorer differentiation [12].

In our cohort, right-sided tumors accounted for the majority of operated mucinous cancers, consistent with earlier reports demonstrating a predilection of MAC for the proximal colon [11,13]. This anatomical distribution likely reflects underlying embryological and molecular differences between right- and left-sided colorectal cancers. The biological divergence between proximal and distal colorectal tumors has become increasingly evident over the last decade. The right colon originates embryologically from the midgut, whereas the left colon and rectum derive from the hindgut. These developmental differences are accompanied by distinct carcinogenic pathways, microbiome composition, immune microenvironments, and genomic profiles. Previous molecular studies have shown that right-sided MACs are more commonly associated with microsatellite instability (MSI), CpG island methylator phenotype (CIMP), deficient mismatch repair, BRAF mutations, and overexpression of mucin-related genes such as MUC2 and MUC5AC [11]. In contrast, left-sided and rectal tumors more frequently demonstrate chromosomal instability pathways and TP53-related carcinogenesis. These biological distinctions may partially explain the survival heterogeneity observed in our study.

Interestingly, despite older patient age and lower rates of chemotherapy utilization, right-sided mucinous tumors demonstrated superior cancer-specific survival compared with left-sided and rectal MAC. Multivariable analysis confirmed significantly worse outcomes for left-sided and rectal tumors compared with right-sided lesions, even after adjustment for stage, grade, treatment, and demographic variables. These findings may appear contradictory to the conventional colorectal cancer literature, where right-sided tumors are often associated with poorer prognosis. However, MAC represents a biologically distinct subgroup. Previous molecular studies have similarly suggested that mucinous tumors harbor unique immune and genomic landscapes that differ substantially according to anatomical location [11].

Rectal mucinous adenocarcinoma demonstrated a distinct clinical profile in our study. Although rectal tumors were more frequently diagnosed at earlier T stages and had the highest recorded use of chemotherapy and radiotherapy, they remained associated with the poorest long-term cancer-specific survival. Because the SEER database does not provide detailed information regarding treatment sequence, neoadjuvant versus adjuvant therapy, treatment regimens, pathological response, circumferential resection margin status, surgical quality, or recurrence patterns, these findings should be interpreted as descriptive associations rather than evidence of treatment effectiveness. Nevertheless, our results are consistent with previous studies reporting that rectal mucinous adenocarcinoma exhibits distinct biological behavior and less favorable oncological outcomes than non-mucinous tumors and mucinous tumors arising in the colon [10,12]. Collectively, these observations raise the possibility that rectal mucinous adenocarcinoma represents a biologically distinct subgroup that may require dedicated risk stratification and should be specifically evaluated in future prospective studies integrating molecular characteristics and treatment-response data.

Our results also align with previous reports suggesting that the prognostic impact of mucinous histology may differ substantially between colon and rectal cancer. Hogan et al. demonstrated improved overall survival in mucinous colon cancer after exclusion of rectal tumors, emphasizing the importance of analyzing colon and rectal cancers separately [14]. Similarly, several population-based studies reported that adverse prognostic implications of mucinous histology are more pronounced in rectal and advanced-stage disease [10,15]. This finding further strengthens the concept that rectal and colon cancers should be considered distinct clinical entities.

Temporal survival analyses additionally suggested gradual improvements in oncological outcomes over recent decades across all anatomical groups. These improvements likely reflect advances in surgical quality, perioperative care, systemic therapy, molecular stratification, and multidisciplinary management. However, the persistent survival disadvantage observed in rectal and left-sided MAC indicates that important challenges remain unresolved.

From a clinical perspective, our findings suggest that anatomical tumor location may serve as an additional prognostic parameter beyond conventional staging systems in mucinous colorectal cancer. Patients with rectal MAC, who demonstrated persistently inferior cancer-specific survival despite intensive multimodal treatment, may benefit from closer postoperative surveillance strategies and individualized treatment pathways. Conversely, the relatively favorable outcomes observed in right-sided MAC support the concept that molecularly distinct biological subgroups may exist within mucinous colorectal cancer, potentially allowing more refined risk stratification approaches.

The present study has several strengths. The use of a large population-based database allowed robust comparison of anatomical subgroups and provided substantial statistical power for survival analyses. Furthermore, separate evaluation of right colon, left colon, and rectum enabled more precise characterization of anatomical heterogeneity within mucinous colorectal cancer. Nevertheless, several limitations should be acknowledged. First, the retrospective nature of the study introduces inherent selection bias. Second, detailed molecular data including MSI, KRAS, BRAF, and CIMP status were unavailable in the SEER database. Third, information regarding specific chemotherapy regimens, total neoadjuvant therapy protocols, surgical quality metrics, and recurrence patterns was limited. Fourth, the SEER database does not provide detailed information on disease recurrence, patterns of metastatic spread, or post-recurrence treatment. Fifth, unmeasured confounders—including performance status, comorbidity burden, surgeon experience, institutional volume, and the extent of multidisciplinary team involvement—may have influenced treatment allocation and survival outcomes in ways that our models cannot account for. Finally, follow-up duration was necessarily shorter for patients diagnosed in the most recent period (2018–2023), which may have affected the stability of long-term survival estimates, particularly the 5-year analyses.

## 5. Conclusions

Mucinous colorectal adenocarcinoma demonstrates substantial heterogeneity according to anatomical tumor location. Right-sided mucinous adenocarcinoma was more prevalent and was associated with more favorable cancer-specific survival, whereas rectal mucinous adenocarcinoma exhibited the poorest long-term oncological outcomes despite higher recorded use of chemotherapy and radiotherapy. These findings suggest that anatomical tumor location should be considered in the prognostic assessment of patients with mucinous colorectal adenocarcinoma and support the need for location-specific risk stratification. Future prospective studies integrating anatomical location with comprehensive molecular and immunological characterization, including MSI, mismatch repair deficiency, KRAS, BRAF, and CIMP status, are warranted to validate these findings and improve individualized prognostic assessment and therapeutic decision-making.

## Figures and Tables

**Table 1:** Baseline demographic and clinicopathological characteristics of operated mucinous colorectal cancer patients by anatomical tumor location.

Variable	Right Colon (*n* = 25,317)	Left Colon (*n* = 10,408)	Rectum (*n* = 4449)	*p* Value
**Patient characteristics**				
Age, median [IQR], years	74.0 [63–82]	68.0 [57–78]	65.0 [54–75]	**<0.001**
Sex, female	14,102 (55.7%)	4766 (45.8%)	1730 (38.9%)	**<0.001**
**Tumor characteristics**				
**AJCC T stage (6th ed.)**				**<0.001**
T1	1162 (4.6%)	820 (7.9%)	414 (9.3%)	
T2	3659 (14.5%)	1225 (11.8%)	734 (16.5%)	
T3	15,988 (63.2%)	6158 (59.2%)	2681 (60.3%)	
T4	4508 (17.8%)	2205 (21.2%)	620 (13.9%)	
**AJCC N stage (6th ed.)**				**<0.001**
N0	15,629 (61.7%)	5947 (57.1%)	2272 (51.1%)	
N1	5914 (23.4%)	2681 (25.8%)	1380 (31.0%)	
N2	3114 (12.3%)	1451 (13.9%)	728 (16.4%)	
Missing/Unknown	660 (2.6%)	329 (3.2%)	69 (1.6%)	
**AJCC Stage Group (6th ed.)**				**<0.001**
Stage I	4015 (15.9%)	1640 (15.8%)	869 (19.5%)	
Stage II	11,610 (45.9%)	4304 (41.4%)	1387 (31.2%)	
Stage III	9692 (38.3%)	4464 (42.9%)	2193 (49.3%)	
**Tumor grade**				**<0.001**
Grade 1 (well differentiated)	2546 (10.1%)	1419 (13.6%)	409 (9.2%)	
Grade 2 (moderately differentiated)	16,267 (64.3%)	6543 (62.9%)	2480 (55.7%)	
Grade 3 (poorly differentiated)	4639 (18.3%)	1477 (14.2%)	759 (17.1%)	
Grade 4 (undifferentiated)	540 (2.1%)	167 (1.6%)	75 (1.7%)	
Missing/Unknown	1325 (5.2%)	802 (7.7%)	726 (16.3%)	
Regional nodes examined, median [IQR]	17.0 [12–24]	15.0 [10–21]	13.0 [9–19]	**<0.001**
Regional nodes positive, median [IQR]	0.0 [0–2]	0.0 [0–2]	0.0 [0–3]	**<0.001**
CEA positive/elevated	3441 (13.6%)	1444 (13.9%)	534 (12.0%)	**<0.001**
**Treatment**				
Chemotherapy	7301 (28.8%)	3929 (37.7%)	3051 (68.6%)	**<0.001**
Radiotherapy	266 (1.1%)	821 (7.9%)	2871 (64.5%)	**<0.001**
Time to treatment, median [IQR], days	17.0 [5–31]	19.0 [8–34]	30.0 [17–47]	**<0.001**

Data are presented as *n* (%) for categorical variables and median [interquartile range] for continuous variables. *p* values were derived from the chi-square test for categorical variables and the Kruskal–Wallis test for continuous variables. Only operated patients are included. **AJCC**: American Joint Committee on Cancer; **CEA**: carcinoembryonic antigen; **IQR**: interquartile range.

**Table 2:** Multivariable Cox proportional hazards analysis evaluating the independent association between anatomical tumor location and survival outcomes.

Outcome	Comparison	*n*	Events	Adjusted HR (95% CI)	*p* Value
**Overall survival**					
Overall survival	Left colon vs. right colon	40,174	25,609	**1.14 (1.10–1.17)**	**<0.001**
Overall survival	Rectum vs. right colon	40,174	25,609	**1.19 (1.12–1.25)**	**<0.001**
**Cancer-specific survival**					
Cancer-specific survival	Left colon vs. right colon	40,174	11,391	**1.33 (1.28–1.39)**	**<0.001**
Cancer-specific survival	Rectum vs. right colon	40,174	11,391	**1.45 (1.34–1.57)**	**<0.001**

Models adjusted for age at diagnosis, year of diagnosis, sex, AJCC stage, tumor grade, receipt of radiotherapy, and receipt of chemotherapy. Reference category: right colon. **HR**: hazard ratio, **CI**: confidence interval.

**Table 3:** Multivariable Cox proportional hazards regression analysis of overall survival and cancer-specific survival in mucinous colorectal cancer.

Variable	HR (95% CI)	*p* Value	Reference Category
**Colon**			
**Overall survival (*n* = 35,725 · Events = 22,947)**			
Age (per 10 years)	**1.66 (1.64–1.69)**	**<0.001**	—
Year of diagnosis (per 5 years)	**0.98 (0.96–0.99)**	**<0.001**	—
Sex: male	**1.31 (1.27–1.34)**	**<0.001**	Female
AJCC stage			
Stage I	**0.83 (0.79–0.86)**	**<0.001**	Stage II
Stage III	**1.56 (1.52–1.61)**	**<0.001**	Stage II
**Cancer-specific survival (*n* = 35,725 · Events = 9765)**			
Age (per 10 years)	**1.28 (1.25–1.30)**	**<0.001**	—
Year of diagnosis (per 5 years)	**0.94 (0.93–0.96)**	**<0.001**	—
Sex: male	**1.21 (1.16–1.26)**	**<0.001**	Female
AJCC stage			
Stage I	**0.51 (0.47–0.55)**	**<0.001**	Stage II
Stage III	**2.38 (2.27–2.50)**	**<0.001**	Stage II
**Rectum**			
**Overall survival (*n* = 4449 · Events = 3105)**			
Age (per 10 years)	**1.43 (1.38–1.47)**	**<0.001**	—
Year of diagnosis (per 5 years)	**0.95 (0.92–0.98)**	0.005	—
Sex: female	**0.84 (0.78–0.91)**	**<0.001**	Male
AJCC stage			
Stage I	**0.56 (0.50–0.62)**	**<0.001**	Stage III
Stage II	**0.79 (0.73–0.86)**	**<0.001**	Stage III
**Cancer-specific survival (*n* = 4449 · Events = 1945)**			
Age (per 10 years)	**1.21 (1.17–1.26)**	**<0.001**	—
Year of diagnosis (per 5 years)	**0.92 (0.88–0.96)**	**<0.001**	—
Sex: female	**0.87 (0.79–0.96)**	0.005	Male
AJCC stage			
Stage I	**0.42 (0.36–0.48)**	**<0.001**	Stage III
Stage II	**0.66 (0.59–0.73)**	**<0.001**	Stage III

**AJCC**: American Joint Committee on Cancer, **CI**: confidence interval, **HR**: hazard ratio. Models adjusted for age at diagnosis, year of diagnosis, sex, AJCC stage, tumor grade, receipt of radiotherapy, and receipt of chemotherapy. Colon and rectum analyzed separately.

## Data Availability

The data presented in this study are available upon request from the corresponding author due to privacy and institutional restrictions related to patient-level clinical data.
